# Mechanism of a Composite Energy Field for Inhibiting Damage in High-Silicon Aluminum Alloy During Micro-Turning

**DOI:** 10.3390/mi16111263

**Published:** 2025-11-07

**Authors:** Jiaxin Zhao, Yan Gu, Yamei Liu, Lingling Han, Bin Fu, Xiaoming Zhang, Shuai Li, Jinlong Chen, Hongxin Guo

**Affiliations:** 1 Jilin Provincial Key Laboratory of Micro-Nano and Ultra-Precision Manufacturing, School of Mechatronic Engineering, Changchun University of Technology, Yan’an Ave 2055, Changchun 130012, China; 2202301042@stu.ccut.edu.cn (J.Z.);; 2 Jilin Provincial Key Laboratory of International Science and Technology Cooperation for High Performance Manufacturing and Testing, School of Mechatronic Engineering, Changchun University of Technology, Yan’an Ave 2055, Changchun 130012, China

**Keywords:** high-silicon aluminum alloy, subsurface damage, composite energy field, molecular dynamics simulation, micro-turning

## Abstract

Composite materials are widely utilized for their excellent properties; however, the mismatch in phase response during processing often induces surface and subsurface damage. While reducing the cutting depth is a common strategy to improve quality, it shifts the material removal mechanism from shear to ploughing–extrusion, which can, in fact, degrade the final surface integrity. Energy field assistance is a promising approach to suppress this issue, yet its underlying mechanism remains insufficiently understood. This study investigates high-silicon aluminum alloy by combining turning experiments with molecular dynamics simulations to elucidate the origin and evolution of damage under different energy fields, establishing a correlation between microscopic processes and observable defects. In conventional turning, damage propagation is driven by particle accumulation and dislocation interlocking. Ultrasonic vibration softens the material and confines plastic deformation to the near-surface region, although excessively high transient peaks can lead to process instability. Laser remelting turning disperses stress within the remelted layer, significantly inhibiting defect expansion, but its effectiveness is highly sensitive to variations in cutting depth. The hybrid approach, laser remelting ultrasonic vibration turning, leverages the dispersion buffering effect of the remelted layer and the localized plastic deformation from ultrasonication to reduce peak loads, control deformation depth, and suppress defects, while simultaneously mitigating the depth sensitivity of damage and maintaining removal efficiency. This work clarifies the mechanism by which a composite energy field controls damage in the micro-cutting of high-silicon aluminum alloy, providing practical guidance for the high-quality machining of composite materials.

## 1. Introduction

High-silicon aluminum alloy (Al-Si binary alloy, 25–70 wt% Si) is extensively employed in electronic packaging substrates, engine cylinder liners, wheels, and gearbox housings owing to its high thermal conductivity, low thermal expansion, excellent wear resistance, high specific strength, high stiffness, and low density (2.4–2.7 g/cm^3^) [1,2]. The Si phase is dispersed within the Al matrix as irregular blocks, allowing the material to be characterized as a particle-reinforced metal-matrix composite (PRMMC) with an Al matrix and Si reinforcement [3]. These characteristics confer outstanding physical properties but also introduce significant machining challenges: the presence of hard, brittle Si particles embedded in a soft, ductile Al matrix [4] can lead to various forms of surface and subsurface damage during cutting [5].

To overcome these machining challenges, numerous studies have sought to improve surface quality through the optimization of process parameters and lubrication techniques. For instance, Jing et al. [6] employed a combination of a genetic algorithm (GA) and orthogonal experiments to optimize milling parameters for Al-50 wt% Si. In a separate study, Jing et al. [7] compared dry milling, supercritical CO_2_ (scCO_2_) milling, and scCO_2_-based minimum-quantity lubrication (scCO_2_-MQL). Their findings demonstrated that scCO_2_-MQL significantly reduces cutting force and temperature, mitigates Si-particle damage, and results in lower surface roughness, attributed to enhanced cooling, lubrication, and penetrative heat transfer capabilities.

Although these optimizations enhance process quality to some extent, they provide insufficient suppression of silicon particle-induced damage. Consequently, the benefits of ultrasonic-assisted machining have garnered increasing attention [8,9,10]. Chen et al. [11] compared conventional cutting with ultrasonic vibration cutting using finite-element simulation and experiments, examining the effects of particle position, cutting path, and process parameters. Their work demonstrated that ultrasonic vibration cutting improves surface integrity and reduces the average cutting force and roughness, attributable to intermittent tool-workpiece separation and periodic reversal of the friction direction. Cui et al. [12], building upon a proposed molecular dynamics model for ultrasonic vibration assisted tensile (UVAT) and a finite element (FE) model incorporating a constitutive correction for SiCp/Al, established that ultrasonic energy lowers dislocation density, promotes stress relaxation and plastic deformation within the Al matrix, and alters the primary material removal mechanism from matrix-tearing shear-band cracking (predominant in conventional cutting) to one dominated by the cracking of fine SiC particles within the shear band, thereby improving the damage mode while maintaining removal efficiency.

An alternative strategy for enhancing machinability involves modifying the material’s microstructure. Guru et al. [13] employed friction stir processing (FSP) to refine the Si-particle size, which resulted in superior machinability compared to the as-cast Al-Si alloy. However, since FSP alters the bulk properties of the material, laser surface remelting has been widely utilized to selectively modify surface properties while minimizing impact on the overall performance [14,15]. For example, Tomida et al. [16] utilized a 5 kW continuous laser to generate a remelted layer on Al-30 wt% Si, significantly reducing the Si-particle size from ~25 μm to ~3 μm and achieving a more uniform distribution within the remelted layer. Fu et al. [17] integrated pulsed laser remelting with non-resonant vibration-assisted grinding. Their approach leveraged nanosecond pulsed remelting to refine Si particles, while the periodic tool-workpiece separation contributed to reduced cutting forces. The combined experimental and molecular dynamics (MD) results elucidated the underlying mechanism and validated the efficacy of this low-damage precision machining strategy for high-silicon aluminum alloy.

Although parameter optimization and energy-field assistance can enhance machining quality, the outcomes remain constrained by tool geometry. Many modeling studies assume an idealized, perfectly sharp cutting edge. However, practical tools require a finite edge radius to maintain sufficient stiffness for withstanding cutting loads [18]. This cutting-edge radius profoundly influences the machining process. A growing body of recent research has focused on this aspect. For instance, Theraroz et al. [19] incorporated the cutting-edge radius into an analytical orthogonal cutting temperature model by distributing discrete linear heat sources along the rounded edge and accounting for heat and velocity-direction changes in global coordinates. Their model provides a more accurate prediction of tool wear evolution compared to models neglecting the edge radius. Similarly, Ellappan et al. [20] employed finite element (FE) analysis to investigate the effects of uncut chip thickness and friction coefficient (COF) on the dead-metal zone (DMZ) and stagnation zone, confirming that the edge arc is a primary source of subsurface damage, particularly under DMZ and micro-cutting conditions.

**Figure 1:**
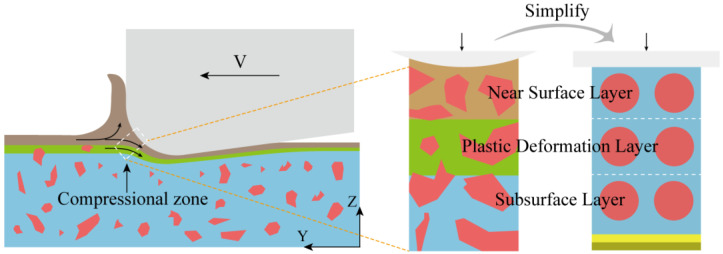
Simulation modeling principle.

During cutting, the cutting edge arc generates significant extrusion in the work-chip separation zone (within the white dashed box in Figure 1). The material on the chip side initiates shear slip and subsequently enters the tool-chip contact zone as cutting progresses. In contrast, the material on the workpiece side remains to form the machined surface and subsurface. Consequently, extrusion from the edge arc plays a critical role in determining surface and subsurface damage. This effect becomes especially pronounced at small cutting depths (below 5 μm), where the size effect causes the arc-shaped compression at the cutting edge to dominate the material removal behavior [21]. Clarifying the damage mechanism induced by cutting-edge arc extrusion in micro-cutting is therefore essential [20]. Utilizing digital image correlation (DIC) and high-bandwidth force measurements, Sodje et al. [18] observed that at low uncut chip thicknesses (<20 μm), a significant size effect occurs—dominated by ploughing–extrusion and grain anisotropy—when the cutting edge sharpness is comparable to the grain size of the workpiece material (e.g., Ti-6Al-4V). This effect leads to strong fluctuations in thermomechanical loads and a fundamental change in chip formation mechanisms. Vollertsen et al. [22] further emphasized the importance of these phenomena by reviewing size effects across various micro-manufacturing processes, concluding that such effects warrant primary consideration under any processing condition.

Existing experimental techniques possess limited capability to resolve micro-cutting mechanisms, particularly when high spatial and temporal resolution is necessary. Under micro-cutting conditions, the governing mechanisms differ substantially from those in conventional cutting, making molecular dynamics (MD) simulation a particularly suitable and prominent investigative tool. MD offers distinct advantages for elucidating microscale processes. Two primary modeling paradigms are commonly employed. The first is reduced-scale, full-system modeling, wherein the actual cutting process is scaled down and a complete system is constructed within the simulation. The results are then interpreted in conjunction with macroscopic observations. For instance, Hao et al. [23] employed MD-based nano-cutting simulations to investigate the wear behavior of nanotwinned cubic boron nitride (nt-cBN) tools on a Ni-Cr-Fe alloy. This approach allows for the concurrent investigation of a wide range of variables and phenomena. The second paradigm is simplified, mechanism-focused modeling, which intentionally minimizes secondary factors to isolate and highlight fundamental processes, thereby clarifying causal relationships. Ren et al. [24], for example, developed simplified models to decouple the effects of extrusion hardening and friction during their study on friction strengthening, significantly improving the clarity of mechanism identification.

The methodological value of molecular dynamics (MD) simulations is particularly pronounced under micro-cutting conditions, a direct consequence of the fundamental differences in material removal mechanisms compared to conventional cutting. At conventional cutting depths, material removal is primarily governed by periodic shear slip. As summarized by Zhao et al. [25], the formation of serrated chips in this regime is primarily explained by three theories: (1) the adiabatic shear band theory, which attributes serration to thermal plastic instability caused by localized heating in the shear zone, leading to concentrated shear deformation; (2) the periodic crack formation theory, which ascribes the serrated morphology to the initiation and propagation of cracks from pre-existing stress concentrations or microvoids; and (3) a combined theory, where adiabatic shear bands initiate first, with cracks subsequently propagating along these bands.

In contrast, when the cutting depth decreases to the micro-scale, the dominant mechanism shifts to a ploughing–extrusion process governed by grain-scale interactions. For instance, Sodje et al. [18] used digital image correlation (DIC) and high-bandwidth force measurements to demonstrate that in Ti-6Al-4V at low uncut chip thicknesses (below 20 µm), a significant “size effect” emerges. This effect, arising when the cutting edge radius is comparable to the material’s grain size, leads to the dominance of ploughing–extrusion and grain anisotropy, resulting in strong fluctuations in thermomechanical loads and a fundamental deviation from conventional chip formation mechanisms. Supporting this, Vollertsen et al. [22] conducted a comprehensive review of size effects in various micro-manufacturing processes, concluding that these effects are a critical consideration across all processing conditions. Given the dominance of such intrinsic, small-scale phenomena, MD simulation is exceptionally well-suited for revealing and qualitatively analyzing the underlying mechanisms in micro-cutting.

In summary, this study addresses two primary challenges in micro-cutting damage analysis: (1) the small scale and diffuse boundaries of the affected zone, combined with the geometric complexity of the cutting-edge arc and material microstructure, which complicate experimental observation and time-resolved analysis; and (2) the prevalence of strong size effects, whereby deformation is governed predominantly by grain-scale mechanisms [18]. To overcome these challenges, we employ molecular dynamics (MD) simulations to investigate the evolution of edge-arc extrusion damage under different energy fields, thereby establishing a foundation for understanding ploughing–extrusion behavior. The MD model isolates and simplifies the extrusion zone beneath the tool’s rounded edge to enhance interpretability, incorporating the following key simplifications: (1) a single-crystal Al matrix containing embedded single-crystal Si particles; (2) spherical Si particles with uniform spacing; (3) application of plane normal pressure via a rigid indenter; and (4) a layered structure dividing the workpiece into near-surface, plastic-deformation, and subsurface regions, following the approach of Xing et al. [26]. Under these assumptions, we simulated the time-dependent mechanical response during compression across different energy-field conditions, with the maximum compression depth set equal to the near-surface layer thickness (Figure 1). The evolution of stress, strain, and defects was then analyzed to elucidate the damage mechanisms in energy-field-assisted micro-cutting.

## 2. Materials and Methods

To investigate surface and subsurface damage in micro-cutting under different energy fields, we performed a comparative experimental analysis of four processes: conventional turning (CT), laser-remelting turning (LRT), ultrasonic vibration turning (UVT), and laser-remelting ultrasonic-vibration turning (LRUVT). Complementary to the experiments, molecular dynamics (MD) simulations were employed to capture the time-resolved microstructural evolution under normal loading. This combined approach aims to elucidate the underlying microscopic damage mechanisms for each energy field, where the role of MD analysis is to provide mechanistic insight rather than to quantitatively replicate experimental results.

### 2.1. Experimental Setup and Procedures

**Figure 2:**
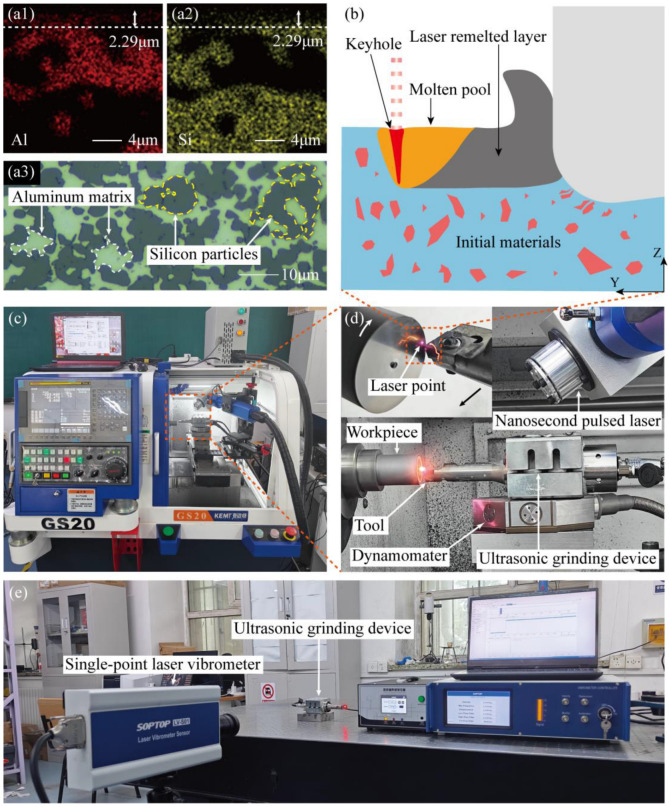
Laser-remelting ultrasonic-vibration turning setup: (**a1**) Subsurface Al distribution after remelting and cooling; (**a2**) Subsurface Si distribution after remelting and cooling; (**a3**) Original microstructure of the high-silicon aluminum alloy; (**b**) Schematic of the composite-field process; (**c**) CNC machining system; (**d**) Machining device and turning details; (**e**) Vibration performance test of ultrasonic device.

The experimental system, depicted in Figure 2, integrates a precision lathe (GS20, KEMT (Changzhou, China)), an ultrasonic vibration apparatus, and a solid-state laser generator (YLPN-Z-ZX350-30, IPG Photonics (Marlborough, MA, USA)) with a working wavelength of 1064 nm. The ultrasonic vibration was driven by a single-actuator 2D planar transducer, a system recognized for its simple structure and drive mode, making it suitable for precision machining of difficult-to-machine materials [27]. The vibration amplitudes, measured using a single-point laser vibrometer (LV-S01, Sunnyinnovation Optical Intelligence. (Hangzhou, China)), were 8 μm in the cutting direction and 6 μm in the depth direction.

The workpiece material was a high-silicon aluminum alloy containing 50 wt% Si, whose original microstructure is shown in Figure 2(a3). Machining was conducted using a polycrystalline diamond (PCD) tool with a 0° rake angle, 7° clearance angle, and a 0.2 mm edge radius. The selection of PCD was based on its high hardness and wear resistance, which are essential for machining abrasive Si particles, and its low chemical affinity to aluminum, which helps suppress tool adhesion and built-up edge formation [28,29,30].

Prior to the turning experiments, preliminary laser remelting tests were conducted to establish parameters that would yield a stable and uniform remelted layer, ensuring its complete removal during subsequent machining. Cubic samples (10 × 10 × 10 mm) were prepared to facilitate cross-sectional analysis. Each workpiece was fixed to the end of a bar, and the laser scanned the end face at a constant linear speed. Following laser treatment, the side of the workpiece was ground flat using a sequence of sandpapers from 600 to 2400 mesh to eliminate potential interference during observation. Subsequently, the ground surface was mechanically polished with a 1 μm diamond suspension to achieve a surface roughness of Ra < 0.05 μm, and then etched with Keller’s reagent to reveal the microstructural features. Elemental distribution in the subsurface region was analyzed using energy-dispersive X-ray spectroscopy (EDS) attached to a scanning electron microscope (SEM). This analysis revealed a distinct separation between the aluminum-silicon mixture in the near-surface region and the underlying material, with the mixed layer exhibiting a consistent depth. Image analysis determined the thickness of this Al-Si solid solution layer to be approximately 2.229 μm, defining the remelted layer thickness (Figure 2(a1,a2)). Consequently, the turning depth was set to 4 μm to operate within the micro-cutting regime while guaranteeing the full removal of the remelted layer.

The turning experiments were then carried out as illustrated in Figure 2. A constant linear speed mode was employed to maintain a uniform surface finish, counteracting the inherent decrease in rotational speed as the tool feeds toward the workpiece center. Surface topography was characterized using a white-light interferometer (NewView 9000, Zygo (Middlefield, CT, USA)), with measurements taken along a straight radial path from the outer edge to the center to minimize random errors. After turning, the workpieces were sectioned by wire cutting to expose the subsurface. The exposed cross-sections were then mounted, ground, polished, etched with Keller’s reagent for 15 s, and finally examined via SEM. The experimental design was structured as a direct comparison of the four machining methods. To ensure a fair comparison, all laser and machining parameters were strictly controlled and kept identical across the relevant conditions. The sole variable between methods was the activation state of the laser and/or ultrasonic vibration, with no other parameter adjustments. The specific parameters, listed in Table 1, were selected based on our prior experimental work [17]. For each processing method, tests were performed on different workpieces from the same material batch using new tools to ensure consistency.

### 2.2. Molecular Dynamics Simulation Methodology

Molecular dynamics (MD) simulation is a powerful tool for investigating microscopic mechanisms [31]. When constructing an MD model, it is crucial to prioritize the core scientific questions and minimize secondary factors that could obscure interpretation. This approach enhances both the clarity of the simulation and the reliability of the resultant conclusions.

In particle-reinforced metal matrix composites (PRMMCs), the mechanical response is predominantly governed by the dispersed reinforcement, and most machining-induced defects are intimately associated with this phase. Consequently, elucidating the interactions among particles and between particles and the matrix is fundamental to understanding the intrinsic processing mechanisms. Guided by this principle, both the Al matrix and the Si particles in our model were represented as single crystals. While polycrystalline structures are more representative of practical materials, simulation outcomes at the MD scale are highly sensitive to geometric variables such as grain size, orientation, and spatial arrangement. This sensitivity can obscure the underlying material behavior. The use of a single-crystal Al matrix with embedded single-crystal Si particles eliminates grain boundary and misorientation effects, thereby better isolating the intrinsic matrix-particle interactions. It is important to note that this simplification does not negate the value of polycrystalline models; rather, it establishes a foundational understanding upon which more complex, realistic simulations can be built once the fundamental relationships are clarified.

The MD model was constructed based on the following simplifying assumptions: (1) both the Al matrix and Si particles are represented as single-crystal phases; (2) Si particles are modeled as spheres with uniform spacing within the matrix; (3) the local loading condition is simplified to plane normal pressure applied by a rigid indenter; and (4) the computational domain is partitioned into surface, subsurface, and matrix layers according to particle depth. The maximum compression depth was confined to the surface layer, enabling analysis of the mechanical response in each stratified region under different processing modes to investigate ploughing–extrusion damage dominated by the cutting-edge arc during micro-turning.

**Figure 3:**
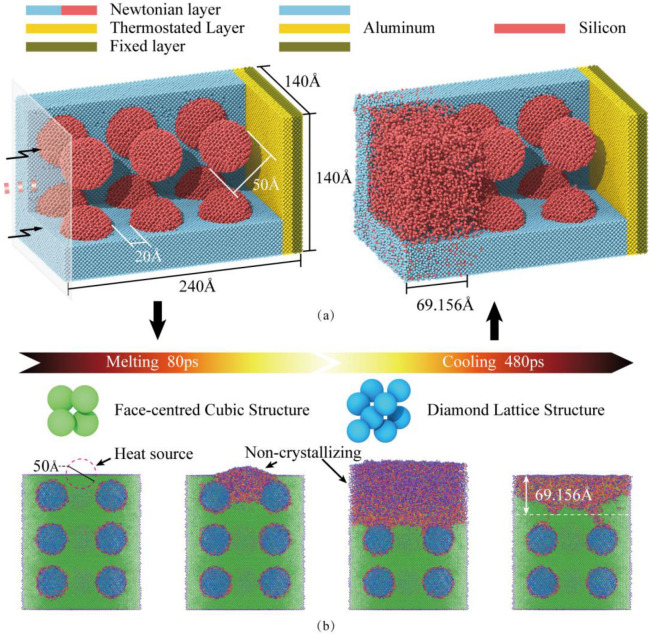
Molecular dynamics simulation methodology. (**a**) MD model for uniaxial compression of high-silicon aluminum alloy: initial configuration (**left**) and structure after laser remelting (**right**). (**b**) Crystal structure evolution during the laser remelting process.

The resulting MD model is illustrated in Figure 3. Simulations were performed using the Large-scale Atomic/Molecular Massively Parallel Simulator (LAMMPS, 2Aug2023-MSMPI), with post-processing conducted in the Open Visualization Tool (OVITO, 3.14.1). The simulation box measures 140 Å × 240 Å × 140 Å and is partitioned along the thickness direction into a Newtonian layer, a thermostat layer, and a fixed boundary layer. The model incorporates twelve spherical Si particles (50 Å diameter, 20 Å nearest-neighbor spacing) uniformly distributed within the Al matrix, totaling 244,246 Al atoms and 39,319 Si atoms (283,565 atoms total), as presented in the left panel of Figure 3a.

Atomic interactions were described using an angular-dependent potential (ADP) [32], which extends the embedded-atom method (EAM) by incorporating dipole and quadrupole terms. The ADP formulation provides improved accuracy for face-centered cubic (FCC) lattice distortion and high-temperature/pressure thermodynamics, making it particularly suitable for simulating laser remelting processes. Prior to compression, the system underwent energy minimization to reach a metastable equilibrium configuration. Periodic boundary conditions were applied along all three axes (X, Y, Z), followed by structural relaxation of the Newtonian and thermostat layers in an NPT ensemble at 300 K to alleviate residual stresses.

For the simulations involving laser remelting (excluding cases of conventional remelting and those using only ultrasonic), after relaxation, the “fix heat” command is used to apply kinetic energy to the atomic regions within the spherical area of the material surface (the laser irradiation zone), simulating the single pulse heating process. The simulated heat source had a radius of 25 Å (model units), with an energy of 943.5 eV applied over an 80 ps pulse duration. Subsequent natural cooling to 300 K occurred over 480 ps. The post-cooling structure (Figure 3a, right) exhibits a solidified Al-Si solution layer approximately 69.156 Å thick. This procedure effectively mimics the rapid melting and solidification induced by a laser pulse. The remelted layer thickness was controlled by adjusting energy density and pulse duration parameters. Note that these simulation parameters were not directly scaled from experimental conditions due to inherent size effects in MD modeling, with the simulation specifically designed to capture the fundamental remelting phenomenon.

Then, the boundary was changed to a non-periodic shrink-wrap boundary, which allows the model to deform freely during compression. Compression was performed in an NVE ensemble. Compression is achieved through a dynamically moving “virtual punch”. This punch is modeled as a rigid plane, whose primary function is to apply a stable displacement load to the workpiece in the Y-axis direction. It is controlled by specifying a time displacement function for the virtual punch (Equation (1)).

(1) $$h(t)=h_{0}-v_{0}t-A\cdot \cos(2\pi t/T),$$

Which $h_{0}$ is the initial height of the indenter, A is the ultrasonic amplitude, When it is 0, it indicates no ultrasonic compression, $v_{0}$ is the compression speed, t is the time, T is the cycle. Detailed simulation parameters are listed in Table 2.

## 3. Results

### 3.1. Experimental Results

**Figure 4:**
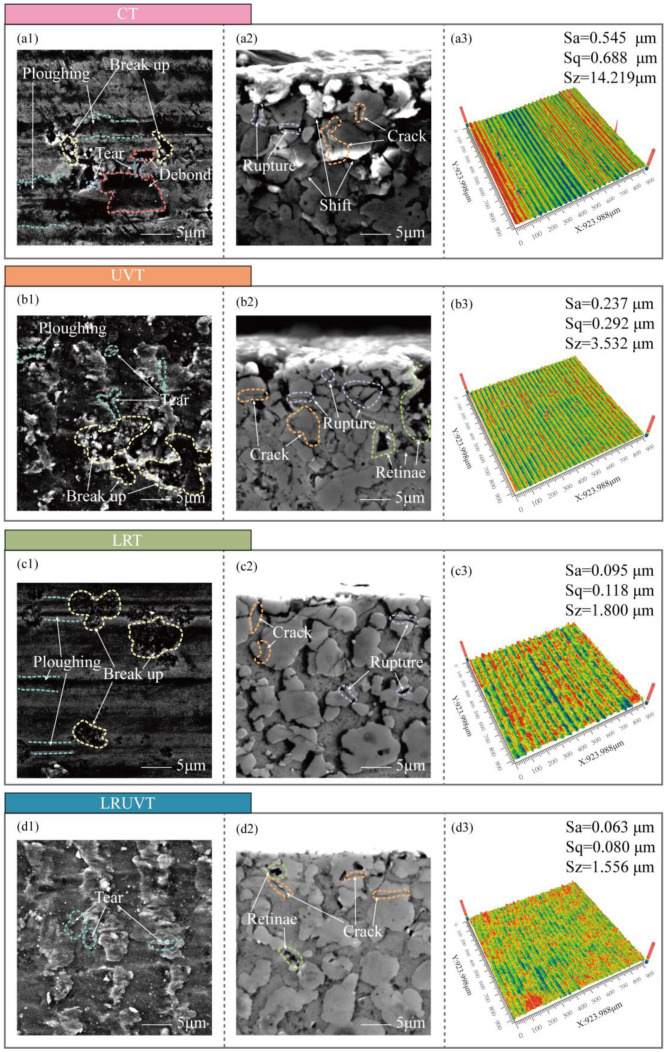
Surface and subsurface integrity under four machining modes: (**a**) CT; (**b**) UVT; (**c**) LRT; (**d**) LRUVT. Panels (**a1**–**d1**) show SEM surface micrographs; (**a2**–**d2**) show SEM subsurface micrographs; (**a3**–**d3**) show Zygo surface topography image.

Figure 4 summarizes the surface and subsurface morphologies for the four machining modes. Surface roughness measurements, taken at 15 evenly spaced points along two perpendicular lines on the end face, yielded average values (Ra) of 0.5336 μm for CT, 0.2304 μm for UVT, 0.0927 μm for LRT, and 0.0642 μm for LRUVT. The representative micrographs shown correspond to the surfaces with roughness values closest to these averages. Conventional turning (CT) resulted in a machined surface marked by extensive particle debonding and fracture, alongside ploughing and tearing grooves proportional to the particle size (Figure 4(a1)). Larger particles were more susceptible to debonding, while smaller particles tended to fracture. Collective particle breakage occurred due to mutual extrusion during ploughing, contributing to pronounced surface non-uniformity. Subsurface analysis (Figure 4(a2)) revealed severe plastic deformation, evidenced by significant displacement of Si particles, reduced inter-particle spacing leading to clustering, and numerous cracks and interfacial defects at the Si/Al boundaries. The compromised matrix support also led to particle pull-out during metallographic preparation. In summary, the CT process generates considerable surface waviness and interfacial damage that extends deep into the substrate.

Ultrasonic vibration turning (UVT) produces periodic vibration marks on the machined surface (Figure 4(b1)). While particle fracture persists and the aluminum matrix exhibits more pronounced tearing, debonding of large particles is substantially reduced. The ploughing grooves caused by fracture or debonding become less frequent and shorter, even as the incidence of surface-particle fracture increases. Subsurface analysis (Figure 4(b2)) reveals a damage layer significantly shallower than that in CT, primarily consisting of fractures in near-surface particles. Few particles were dislodged during sample preparation, and the pronounced particle displacement and clustering characteristic of CT are seldom observed. The corresponding surface roughness was 56.82% lower than CT, indicating that UVT effectively confines material removal to the surface layer—predominantly through particle fracture and matrix tearing—thereby minimizing deep material displacement and interfacial damage. These results indicate that UVT localizes material removal to the surface layer (mainly surface-particle fracture and matrix tearing), which greatly weakens deep displacement and interfacial damage.

In contrast, laser-remelting turning (LRT) produces a markedly smoother surface (Figure 4(c1)). Although localized particle fracture persists, the fractures are shallower and more contained, resulting in thinner and shallower associated ploughing traces. The subsurface (Figure 4(c2)) shows a notable absence of severe defects, with only occasional interfacial cracks and isolated particle fractures visible at the Si/Al interfaces. This method reduced the surface roughness by 82.63% compared to CT, demonstrating its efficacy in suppressing surface waviness and serious subsurface damage, thereby restricting imperfections to a shallow, mild region.

Laser-remelting ultrasonic-vibration turning (LRUVT) yields the most favorable combination of surface and subsurface integrity (Figure 4(d1,d2)). While faint vibration marks remain, they are substantially shallower. Matrix tearing, commonly associated with UVT, is strongly suppressed, and particle debonding is nearly eliminated. Pits originating from particle fracture are also scarce. Although the visual texture may appear slightly less uniform than LRT, LRUVT achieves the lowest measured surface roughness, attributable to effective suppression of particle fracture and consequent ploughing (72.13% and 30.74% lower than UVT and LRT, respectively). The subsurface microstructure remains largely continuous, with a dramatic reduction in crack density and no evidence of severe localized damage. Overall, LRUVT successfully confines damage to an extremely shallow surface layer, strongly suppressing tearing, debonding, and pitting to deliver the minimal subsurface defect density and optimal surface roughness among all methods investigated.

### 3.2. Simulation Results

**Figure 5:**
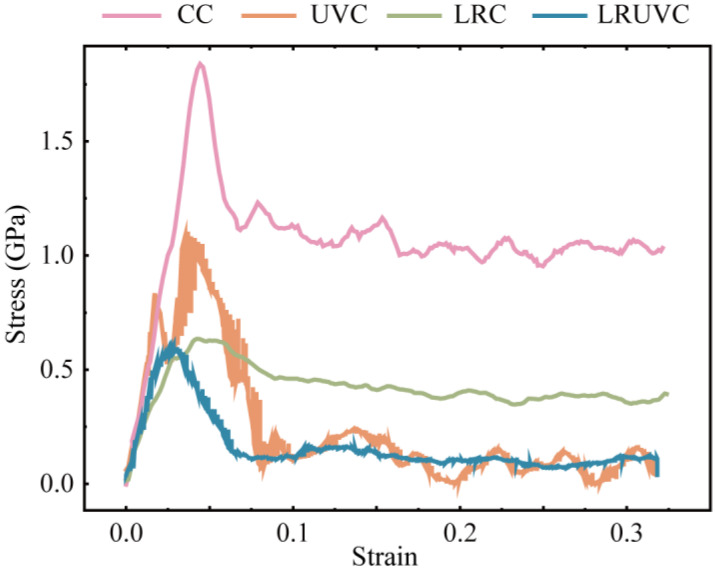
Representative stress-strain curves for four compression modes.

The overall mechanical response under each processing mode was compared through the calculated stress–strain curves presented in Figure 5. Conventional compression (CC) exhibits the highest yield peak stress, followed by a high early-stage flow stress plateau with irregular fluctuations, consistent with continuous loading and pronounced work hardening. In ultrasonic vibration compression (UVC), the stress reaches an initial peak before rapidly decreasing to a lower, periodically oscillating level, characteristic of intermittent loading and demonstrating significant acoustic softening [33]. Laser-remelting compression (LRC) shows substantially reduced peak and steady-state flow stresses compared to CC. The stress attenuates gradually during compression, maintaining a moderate level with limited work hardening and no distinct yield point phenomenon. Laser-remelting ultrasonic-vibration compression (LRUVC) displays the lowest initial peak stress, which then rapidly decays to the minimum sustained stress level with only minor oscillations. Collectively, both laser remelting and ultrasonic vibration effectively reduce peak and average stress, with the LRUVC mode providing the most pronounced and stable suppression of both stress magnitude and fluctuations.

Four characteristic compression depths common to all modes were identified from the stress–strain curves for detailed analysis: 6 nm (elastic stage), 20 nm (early plastic stage, termed ‘pre-compression’), 35 nm (mid-compression, ~50% of near-surface thickness), and 80 nm (end of compression, full near-surface thickness).

**Figure 6:**
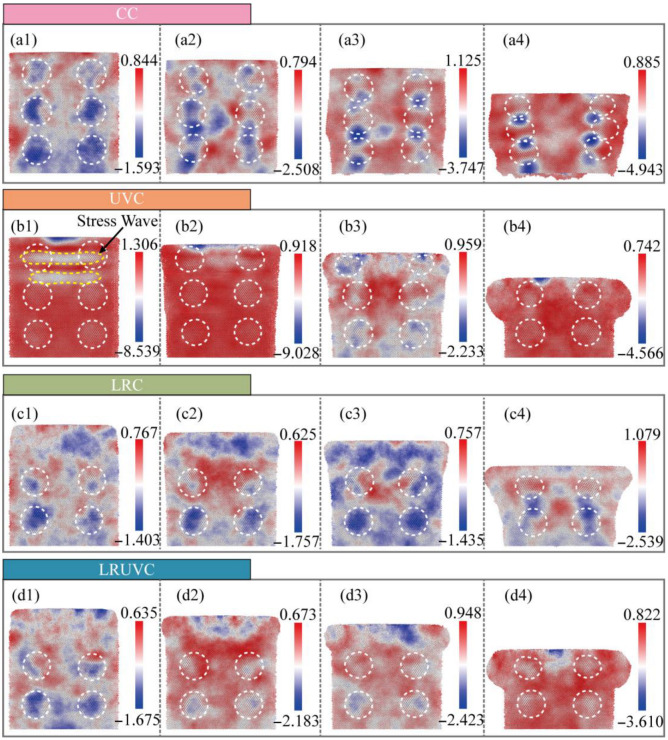
Snapshots of Hydrostatic stress evolution (GPa) for four compression modes (positive = tension, negative = compression). The white dotted line represents the outline of the silicon particles. Panels (**a**–**d**) correspond to CC, UVC, LRC, and LRUVC; panels (**1**–**4**) correspond to 6 nm, 20 nm, 35 nm, and 80 nm.

Figure 6 presents the evolution of hydrostatic stress, a key parameter governing volumetric changes and exerting a strong influence on defect formation and crack propagation. Figure 6a–d represent the compression modes CC, UVC, LRC, and LRUVC, while columns (1–4) correspond to depths of 6, 20, 35, and 80 nm, respectively.

During the initial compression stage in CC (Figure 6(a1)), the overall hydrostatic stress is low. Stress concentrations are primarily localized near silicon particles, with a notably higher magnitude at the bottom boundary, indicating a downward propagation trend that is ultimately limited by the model dimensions At the early compression stage (Figure 6(a2)), hydrostatic stress increases with the emergence of work hardening. High compressive stress transfers to the particle extremities and the intervening Al matrix, forming continuous high-stress bands. During the middle stage (Figure 6(a3)), the stress intensifies and becomes increasingly concentrated in the interparticle regions and at the fixed-layer interface. This progression culminates at the final compression stage (Figure 6(a4)), where the stress is intensely localized at the contact points between clustered particles.

The initial stage of UVC (Figure 6(b1)) exhibits the highest instantaneous hydrostatic stress among all modes, with a peak below −11 GPa. The ultrasonic excitation generates a surface high-pressure zone that propagates downward as a stress wave. This wave attenuates rapidly, resulting in a shallower affected depth than in CC despite the higher initial stress peak. In the early UVC stage (Figure 6(b2)), the peak stress decreases, manifesting as acoustic softening through a reduction in the effective elastic modulus. Although the stress wave persists, material yielding occurs, and the diminished modulus restricts deeper penetration. By the middle stage (Figure 6(b3)), further material softening in the top region causes the stress peak to fall below the CC level at equivalent depth. A localized high-stress zone develops near the end face and undergoes in-situ relaxation, accompanied by the near-complete dissipation of the stress wave. In the final stage (Figure 6(b4)), a subsequent loading cycle initiates as deeper particle layers engage, producing a renewed stress increase with a diminished peak attributable to cumulative softening effects.

The mechanical response in LRC demonstrates a distinct stress distribution. The stress field remains dispersed and maintains a low magnitude with minimal increase throughout the early to middle stages (Figure 6(c1–c3)), primarily concentrating within the remelted layer and around the bottom particles. The disordered microstructure of the remelted layer facilitates load distribution, resulting in an overall stress level comparable to the initial CC stage and confirming the stress-reduction effect of laser remelting. This behavior persists until the final compression stage (Figure 6(c4)), when the underlying particle layer begins to influence the mechanical response, producing a hybrid LRT-CC mode. The stress magnitude progressively rises, and the concentration zone transitions toward the inter-particle aluminum matrix. With continued compression, the stress distribution converges toward the CC pattern. Consequently, effective utilization of laser remelting in practice requires precise matching of the cutting depth to the remelted-layer thickness.

The LRUVC exhibits a transitional response, evolving from a remelting-dominated to an ultrasonic-dominated state. At the initial compression stage (Figure 6(d1)), the overall stress distribution closely resembles that of LRC (cf. Figure 6(c1)), with concentrations within the remelted layer and bottom particles. The superimposed ultrasonic energy elevates the stress magnitude slightly above pure LRC levels, although it remains substantially lower than in UVC. During the early to middle compression stages (Figure 6(d2,d3)), the stress magnitude experiences a moderate increase. The system undergoes a rapid transition from the initial remelting-dominated regime to a state exhibiting ultrasonic-vibration characteristics, achieving this shift more rapidly than in pure UVC. Crucially, the hybrid mode eliminates the pronounced early stress peak and deep stress wave propagation characteristic of UVC—phenomena that pose practical risks by potentially activating pre-existing defects. Instead, LRUVC maintains a moderated initial response while directly accessing the more stable mid-to-late-stage deformation behavior of UVC. By the final compression stage (Figure 6(d4)), load-bearing transitions predominantly to the underlying unremelted layer. The resulting stress distribution pattern resembles that of UVC but exhibits a moderately reduced magnitude compared to pure UVC (cf. Figure 6(b4)). With continued compression, the response would converge completely toward the characteristic UVC state.

In summary, CC produces deep hydrostatic stress penetration with intense sub-surface concentration and severe deformation. Ultrasonic vibration compression (UVC) localizes stress near the surface but generates excessive early-cycle peaks and potentially detrimental stress waves. Laser-remelting compression (LRC) effectively mitigates stress concentration but provides limited control over its penetration depth. The hybrid LRUVC mode achieves optimal performance, maintaining low stress peaks and uniform distribution throughout loading, thereby demonstrating superior capabilities in stress regulation and mitigation.

**Figure 7:**
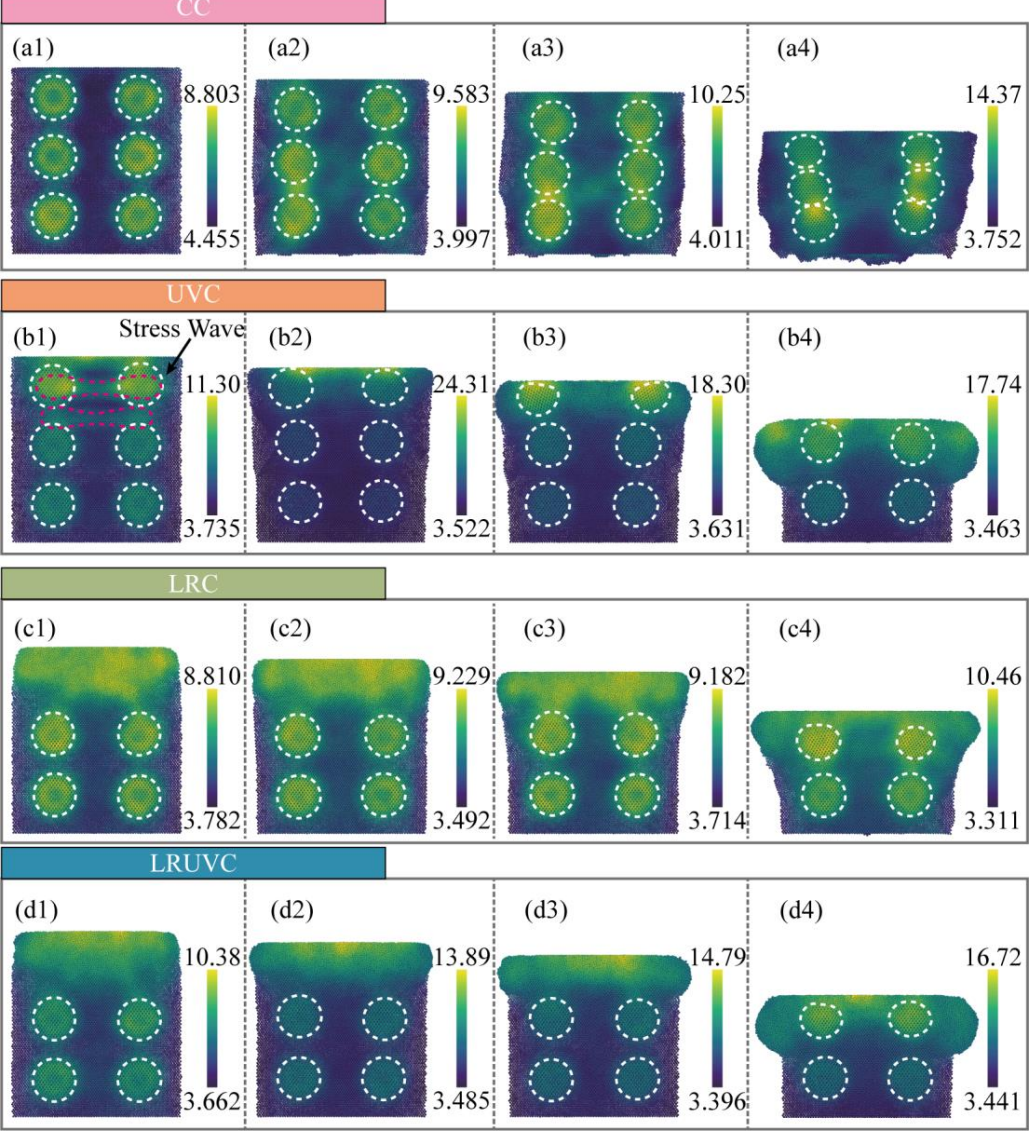
Snapshots of von Mises stress evolution (GPa) for four compression modes (positive = tension, negative = compression). The white dotted line represents the outline of the silicon particles. Panels (**a**–**d**) correspond to CC, UVC, LRC, and LRUVC; panels (**1**–**4**) correspond to 6 nm, 20 nm, 35 nm, and 80 nm.

Von Mises stress, which characterizes distortion energy and serves as a fundamental yield criterion, is analyzed in Figure 7. During the initial stage of conventional compression (CC, Figure 7(a1)), prior to yielding onset, the stress field exhibits a diffuse distribution. Beyond localized concentrations surrounding Si particles, the stress is widely distributed throughout the workpiece without pronounced localization or directional propagation. As compression progresses (Figure 7(a2)), the von Mises stress increases substantially. Silicon particles distributed along the deformation path serve as nucleation sites for stress propagation along slip systems. This behavior reflects the combined effects of load-transfer strengthening and work hardening, both contributing to the elevated von Mises stress levels. With continued compression (Figure 7(a3)), the von Mises stress intensifies and becomes increasingly concentrated at particle contact points. This progression culminates in the final stage (Figure 7(a4)), where the stress reaches its maximum magnitude and becomes intensely localized within interparticle contact regions.

In UVC, the von Mises stress similarly exhibits high magnitude and localized concentration. At the initial stage (Figure 7(b1)), prior to yielding onset, the primary load is carried by the first particle layer and the adjacent aluminum matrix [34]. A distinct stress wave emerges in the von Mises distribution, spatially coincident with the hydrostatic wave, demonstrating a coupled compression-shear transmission mechanism governed by the crystal lattice and loading path. As compression progresses (Figure 7(b2)), the stress wave attenuates while the near-surface region supports the predominant von Mises stress. The stress magnitude increases abruptly, transitioning the material into an intense compression-shear state. In contrast to the hydrostatic stress evolution (cf. Figure 6(b3,b4)), the von Mises stress exhibits only moderate attenuation during continued loading, indicating sustained accumulation of distortion energy through persistent high-level fluctuations rather than periodic decay.

In LRC, the initial stage (Figure 7(c1)) exhibits a remelted layer characterized by a disordered solid solution structure instead of a regular crystal lattice. This amorphous structure inhibits the formation of localized stress concentrations, resulting in a stress field of reduced magnitude and enhanced uniformity. During the early compression stage (Figure 7(c2)), while the stress magnitude increases, it maintains a dispersed distribution throughout the remelted layer rather than concentrating at particle-matrix interfaces. As compression progresses to greater depths (Figure 7(c3)), the stress distribution shifts toward the interfaces between the unremelted matrix and embedded particles. While this propagation trend resembles that observed in CC, the peak stress magnitude remains substantially lower, demonstrating the remelted layer’s capacity to mitigate subsurface shear stress transmission. In the final stage (Figure 7(c4)), load transfer occurs primarily to the underlying unremelted particle layer. Although the resulting stress pattern converges toward the CC distribution and the stress level increases correspondingly, the degree of localization remains significantly attenuated compared to conventional compression.

In LRUVC, the von Mises stress shows the synergy of remelting and ultrasonic. At the start (Figure 7(d1)), the pattern is close to LRC. The remelted layer lowers the peak and greatly eases the transient stress wave from the ultrasonic. The stress is concentrated at the surface and the first particle layer. As depth increases, the stress rises and focuses at the top. But there is no sharp peak as in UVC. The change is smoother. It means that the remelted layer dissipates the early strong wave and prevents deep subsurface distortion. By the end (Figure 7(d4)), the remelted layer has flowed outward and lost load-bearing capacity. So, the pattern approaches UVC. The overall peak remains slightly lower than in the pure UVC case.

Summary, in CC, von Mises stress rises with compression and concentrates at particle contacts, reflecting load transfer and work hardening. In UVC, a clear early stress wave appears with strong compression-shear coupling. Although the wave fades later, the von Mises level stays high, showing continued build-up of distortion energy. In LRC, the remelted layer weakens concentration and helps dissipate distortion energy. So, the overall level is lower and more uniform. With deeper loading, the pattern moves toward CC. In LRUVC, remelting and ultrasonic work together. The dispersion effect of the remelted layer suppresses the stress wave effect caused by vibration and reduces the peak stress, so that the stress field remains uniform. The stress concentration effect of ultrasonics prevents the stress caused by the remelting layer from being too dispersed and concentrates the stress on a more “surface” position.

The equivalent plastic strain (EPS) is computed from the deformation gradient tensor of local atomic neighbors. As a stress-driven response, it captures both elastic deformation and irreversible plastic rearrangement. Its values can span several orders of magnitude. To ensure comparison across modes and loading stages, a single colormap was used for all EPS maps. The lower limit is 0.002 to suppress background noise and small numerical fluctuations. The upper limit is 1.5. It covers the main strain ranges of the elastic stage (von Mises strain < 0.02), dislocation slip and twin initiation stage (0.05–0.3), local amorphization and severe plastic deformation stage (0.5–1.0). A small number of values above the upper limit are shown by truncation (clipped to the maximum color), which avoids compressing the overall color dynamic range.

**Figure 8:**
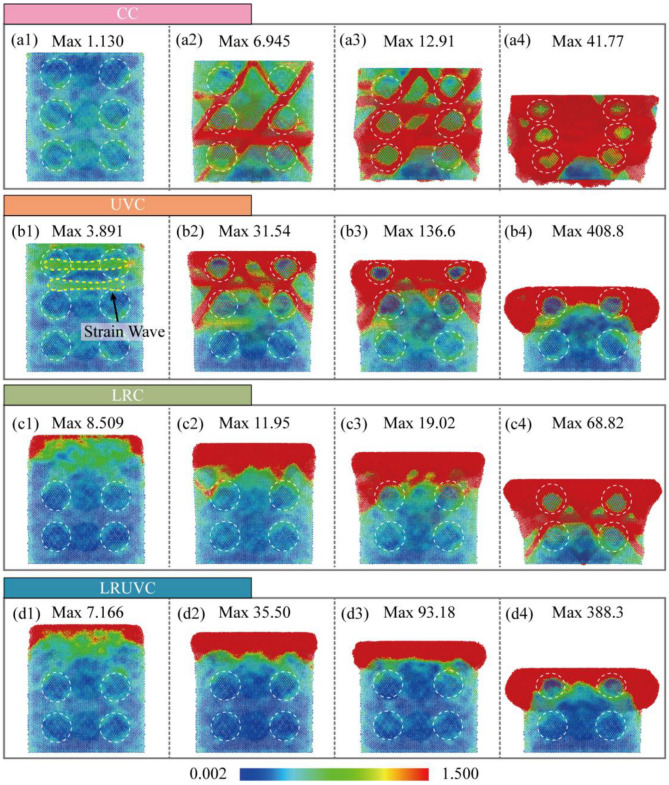
Snapshots of EPS evolution for four compression modes. The white dotted line represents the outline of the silicon particles. Panels (**a**–**d**) correspond to CC, UVC, LRC, and LRUVC; panels (**1**–**4**) correspond to 6 nm, 20 nm, 35 nm, and 80 nm.

At the initial stage of CC (Figure 8(a1)), the strain value is at a low level, the overall distribution is dispersed, and there is no obvious concentrated area, which reflects the uniform deformation response of the material in the elastic stage. In the intermediate stage (Figure 8(a2)), the strain rises rapidly with the increase in von Mises stress. A high-strain band is formed at the slip surface between the particle and the matrix, showing the crack initiation along the interface and slip surface. At the middle stage of compression (Figure 8(a3)), the strain is further increased. And it is highly concentrated between the particles and near the fixed layer. The local plastic zone expands significantly. The plastic strain of the matrix is wrapped around the particles to slide together. The high strain of the interface shows the phenomenon of particle debonding. At the end of compression (Figure 8(a4)), the strain level reaches the maximum. A wide range of high-strain areas is distributed between the particles and the central region. The workpiece has significant pier coarse deformation. The particles are significantly displaced and accumulated. The high plastic strain caused by particle accumulation easily leads to the occurrence of particle fracture. The conventional compression shows the law of uniformity in the early stage, gradual development in the middle stage, and finally comprehensive expansion. The matrix slips and wraps the accumulation of particles, and the plastic distortion is mainly regulated by the coordination of particles and matrix.

At the initial stage of UVC (Figure 8(b1)), EPS is relatively high and is concentrated mainly at the surface of the remelted layer. The overall pattern remains fairly uniform. The disordered structure has limited long-range support. So, it is more prone to strain. In the early stage (Figure 8(b2)), EPS rises quickly, and the affected area expands. While the high strain is still mostly confined to the top of the remelted layer. In the middle stage (Figure 8(b3)), EPS keeps increasing, though still at a moderate level. The distribution lies mainly within the remelted layer and begins to extend downward. At the end stage (Figure 8(b4)), EPS increases further, and the pattern gradually approaches CC. High-strain zones extend downward along slip planes, indicating a higher risk of cracking in the matrix beneath the remelted layer. Overall, loading after remelting shows a high initial level, slow accumulation, locally dispersed distribution, and delayed diffusion, reflecting the homogenizing and buffering effects of the remelted layer on plastic deformation. However, protection of the underlying region gradually fails at later stages.

At the initial stage of LRC (Figure 8(c1)), EPS is relatively high and is concentrated mainly at the surface of the remelted layer. The overall pattern remains fairly uniform; the disordered structure has limited long-range support. So it is more prone to strain. At the early stage (Figure 8(c2)), EPS rises quickly. And the affected area expands. The high strain is still mostly confined to the top of the remelted layer. At the middle stage (Figure 8(c3)), EPS keeps increasing. Though it is still at a moderate level. The distribution lies mainly within the remelted layer and begins to extend downward. The end stage (Figure 8(c4)), EPS increases further, and the pattern gradually approaches CC. High-strain zones extend downward along slip planes, indicating a higher risk of cracking in the matrix beneath the remelted layer. Overall, after loading ultrasonic, it shows the characteristics of low initial, rapid increase, local concentration, and extreme peak. The occurrence of local plastic failure is significantly accelerated, which reflects the homogenizing and buffering effects of the remelted layer on plastic deformation. However, protection of the underlying region gradually fails at later stages.

In LRUVC, EPS reflects the synergy between remelting and ultrasonic vibration. At the initial stage (Figure 8(d1)), the strain level is high and the distribution reaches slightly deeper than in LRC. Yet, the concentration still lies near the top of the remelted layer and around the first particle layer, indicating that the remelted layer effectively weakens the near-surface concentration caused by ultrasonic impact. In the early and middle stages (Figure 8(d2,d3)), EPS rises rapidly and the affected region expands. However, the growth rate is lower than in UVC because of the remelted layer. And the depth of the ultrasonic concentration effect is smaller than in LRC, demonstrating the complementary advantages of the hybrid field. This ability to hold a more stable strain within a smaller region combines efficiency with stability. At the end stage (Figure 8(d4)), the overall pattern approaches UVC. The peak is slightly lower. The edge localization is milder. And the end-stage failure seen in LRC does not appear. Overall, the hybrid mode couples the strengths of both fields and controls both strain intensity and penetration depth throughout loading, highlighting the dual benefits of fast effectiveness and process stability in LRUVC.

Overall, CC shows strain concentrated along slip planes at the particle-matrix interface, indicating a plastic-deformation mode dominated by particle debonding and particle accumulation. UVC exhibits strong concentration with extreme peaks. Although it accelerates strain development and limits penetration depth, the mid-cycle response is prone to instability, leading to particle fracture and surface spalling. At the early stage of LRC, strain is higher yet more uniform, reflecting the homogenizing and buffering roles of the remelted layer in plastic flow. However, the response inevitably shifts toward CC with deeper loading. LRUVC combines the strengths of both. It suppresses the severe early ultrasonic strain concentration while keeping peaks lower and the distribution controlled. As a result, it balances fast response with stability and provides the best control over the evolution of plastic strain.

**Figure 9:**
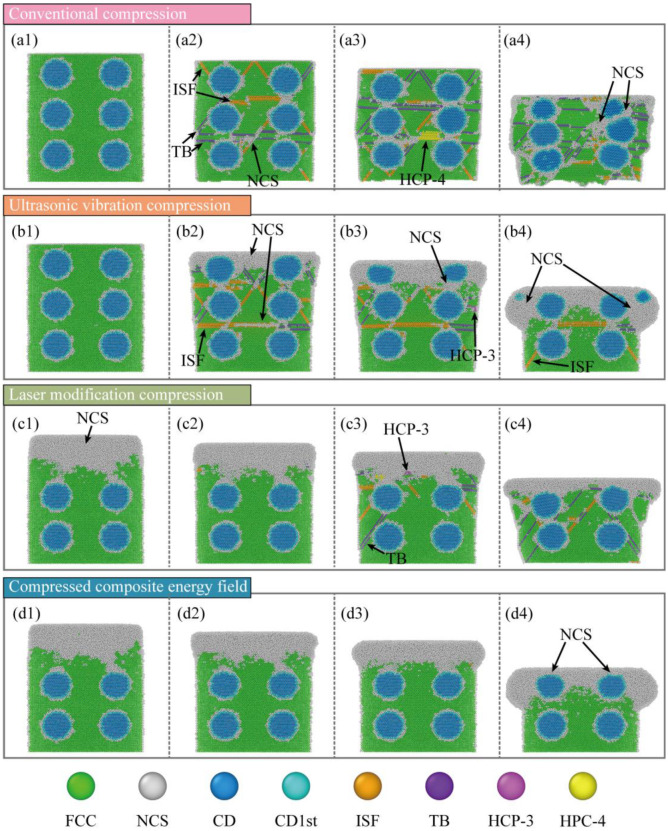
Snapshots of defect evolution for four compression modes. Panels (**a**–**d**) correspond to CC, UVC, LRC, and LRUVC; panels (**1**–**4**) correspond to 6 nm, 20 nm, 35 nm, and 80 nm.

To probe the microscopic slip mechanisms during compression, the Crystal-Analysis-Tool (CAT) was used to identify and count defects. Figure 9 shows the evolution of the defect distributions. In CC, the workpiece with only a few defects at interfaces is mainly face-centered cubic (FCC) in the early stage (Figure 9(a1)). At the next stage (Figure 9(a2)), stacking faults grow rapidly along slip planes and interfaces. And twins appear in high-strain zones, indicating slip activation. At the middle stage (Figure 9(a3)), defects increase further, and local body-centered cubic (BCC) regions emerge. Multiple defect types form an interwoven network, showing entry into a severe plastic-flow regime. At the end stage (Figure 9(a4)), defects spread across the workpiece. SFs and twins extend over large areas. And overall crystal integrity is seriously degraded.

In UVC, FCC lattice still dominates at the start (Figure 9(b1)), which is similar to CC. At the early stage (Figure 9(b2)), surface crystals within high-strain zones lose long-range order and transform into an amorphous state. At the same time, many stacking faults (SFs) form rapidly between particles and in the subsurface layer. And twin bands appear locally. Some twins further convert to amorphous, evidencing severe plastic flow driven by ultrasonic impact. At the middle stage (Figure 9(b3)), amorphous regions keep expanding downward. But part of the amorphous phase reverts to SFs as the hydrostatic stress decreases. A smaller population of SFs continues to extend to deeper layers. At the end stage (Figure 9(b4)), the top particles and matrix are severely damaged. Large areas of the crystal transform into amorphous material. It is accompanied by a smaller number of defects extending further downward.

In LRC, the surface layer is a disordered solid solution at the initial stage (Figure 9(c1)). And it gradually transitions downward to the FCC lattice. In the early stage (Figure 9(c2)), a small number of SFs form beneath the remelted layer. But their population remains limited. In the middle stage (Figure 9(c3)), defects increase markedly, and the response shifts toward CC. SFs and twins initiate from the remelted layer and from interfaces. In CC and UVC, defect development extends not only along the main FCC slip system {111} (about 45°) but also activates many secondary slip systems (e.g., {100}) due to cross-slip and high hydrostatic stress. After laser remelting, this broader activation is suppressed. At the end stage (Figure 9(c4)), defects continue to grow and interweave. The active slip-system set is narrower. Overall defect content is higher than in UVC yet lower than in CC.

In LRUVC, defect evolution reflects the coupling of the two mechanisms. Throughout loading (Figure 9(d1–d4)), crystal integrity remains very high outside the remelted layer. The strong localization of the high-strain zone keeps structural damage confined. Lattice collapse occurs mainly within this zone. And only a small number of defects form and propagate downward. Although this ideal behavior is partly related to the single-crystal modeling simplification, it still demonstrates the clear advantage of the composite energy field in damage control. This phenomenon was also verified in the wear resistance experiment of Fu et al. [35].

**Figure 10:**
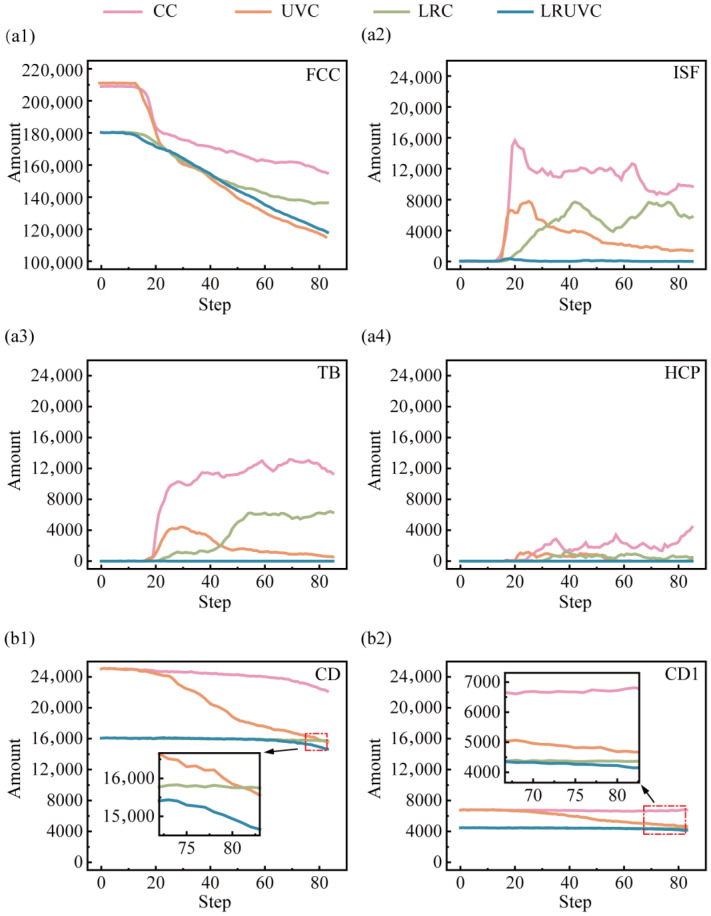
Quantitative evolution of crystal structures under different compression modes as a function of depth. Panels (**a1**–**a4**) plot the fractions of FCC, ISF, TB, and HCP, respectively. Panels (**b1**,**b2**) show the counts of the perfect cubic diamond structure and the first-nearest-neighbor cubic diamond structure.

To clarify defect evolution with increasing compression, Python (3.11.5) was used to extract counts of the relevant defect types and diamond structures. And plotted the results in Figure 10. In Figure 10(a1), the fraction of FCC decreases rapidly at the early stage of compression. The CC and UVC have the fastest drop. By contrast, the remelted layer preferentially bears the load in LRC and LRUVC. So, FCC weakening is more moderate. In Figure 10(a2), intrinsic stacking fault (ISF) counts rise quickly in CC and UVC. Then it declines after reaching a peak, indicating that part of the ISF population transforms into an amorphous phase. Figure 10(a3) shows that the twin boundary (TB) increases markedly and then decays in UVC. It is a trend consistent with the ISF-to-amorphous transition. In CC, however, TB continues to grow after an early rapid rise, indicating delayed weakening. The behavior also appears in LRC. Though it is milder. Figure 10(a4) indicates that HCP accumulates gradually in CC and LRC. While it appears mainly at the mid-stage in UVC. It signals the highly unstable plastic state there. Across these three defect types (ISF, TB, HCP), LRUVC shows very low levels, underscoring the strong defect-control capability of the composite energy field.

Figure 10(b1,b2) presents the evolution of the perfect cubic diamond structure and the first-nearest-neighbor cubic diamond structure. During compression, the perfect diamond count decreases rapidly under UVC, indicating damage to particle integrity. At the late stage of CC, the rate of change of the perfect diamond metric points to a non-negligible level of particle damage. Interestingly, the first-nearest-neighbor diamond metric in CC shows a rebound at later stages, suggesting that under high stress, the diamond framework undergoes not only brittle fracture but also local slip with the formation of stacking faults or twins. The behavior was also reported in Nie [36].

Overall, defects nucleate quickly and spread widely in CC. Stacking faults and twins form in large numbers. And crystal integrity is severely degraded. UVC drives strong early structural evolution, rapidly producing amorphous regions and stacking faults. Twin bands propagating downward. Although it accelerates plastic flow, it also causes severe structural damage. LRC effectively suppresses early defect formation and limits slip-system activation, showing the delay and buffering effects of the remelted layer. However, the response gradually shifts toward CC with deeper compression. LRUVC offers the best defect control. Overall, crystal integrity remains high. And only limited defects evolve. The defects are confined to local high-strain zones. Counts of stacking faults, twins, and amorphous regions are much lower than in the other modes, highlighting the advantage of the composite energy field in damage suppression and structural-integrity maintenance.

**Figure 11:**
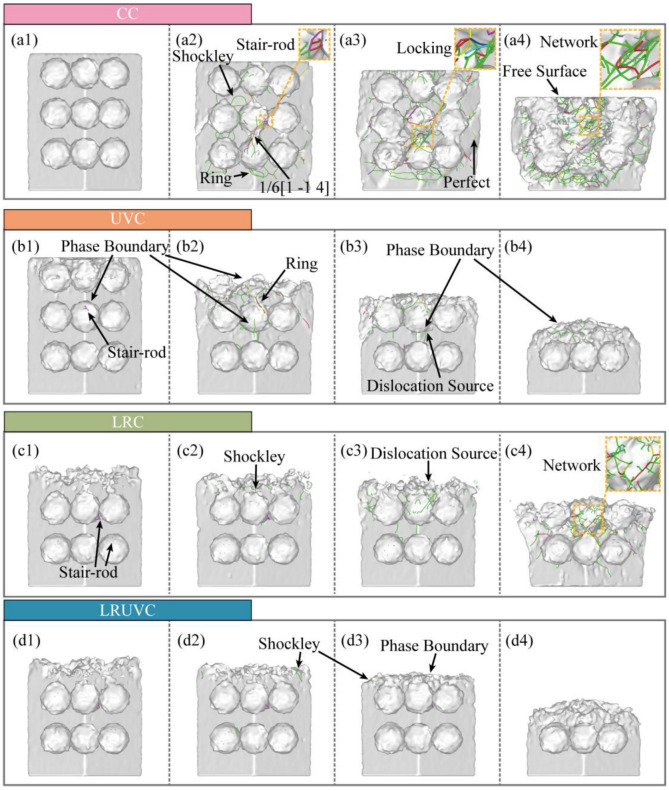
Snapshots of dislocation evolution for four compression modes. Panels (**a**–**d**) correspond to CC, UVC, LRC, and LRUVC; panels (**1**–**4**) correspond to 6 nm, 20 nm, 35 nm, and 80 nm.

**Figure 12:**
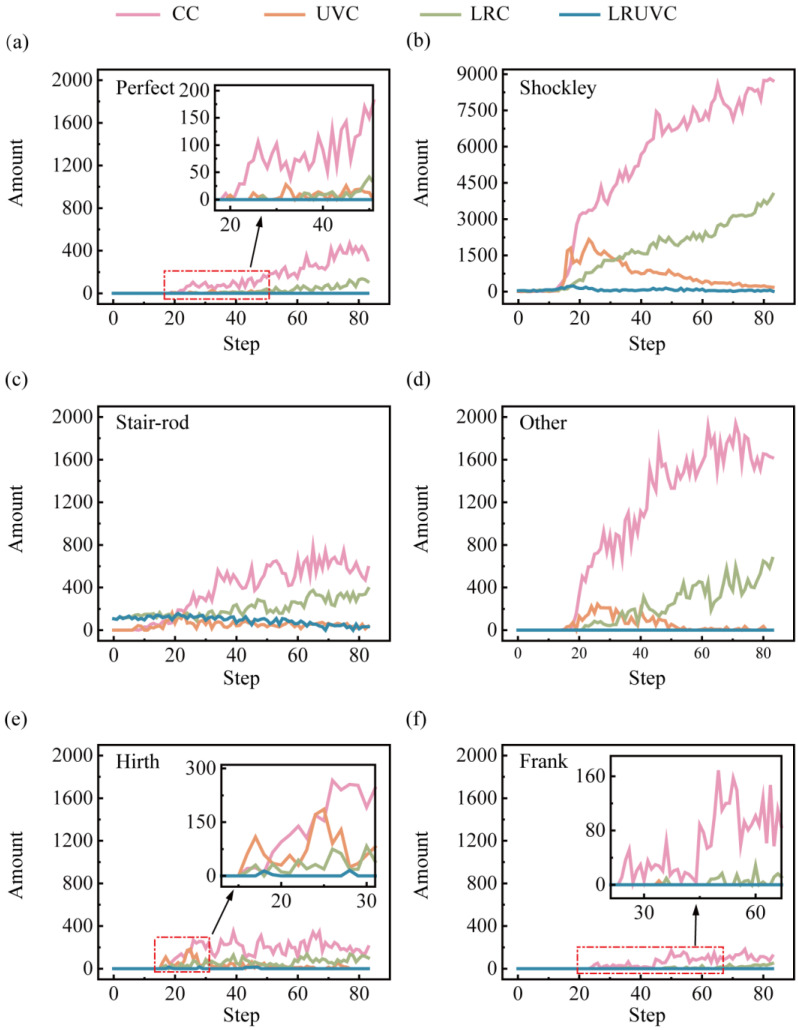
Dislocation-length evolution by type. Panels (**a**–**f**) show perfect, Shockley, stair-rod, other, Hirth, and Frank dislocations, respectively.

Dislocation-evolution snapshots obtained with the Dislocation Extraction Algorithm (DXA) of the different compression modes are shown in Figure 11. The spatial patterns of dislocations are broadly consistent with the defect maps. To clarify how defects develop with compression, it was extracted that the total dislocation length by type was extracted and plotted in Figure 12.

In Al alloys with high stacking-fault energy (SFE), plasticity relies mainly on perfect dislocations and their decomposition products. Perfect dislocations are commonly activated by Frank-Read sources. And the dislocations also nucleate at stress concentrators such as interfaces, holes, particle sharp corners, or grain boundaries. They carry most of the slip and load transfer, and their continuous build-up causes work hardening. Under strong shear or interface constraints, a perfect dislocation can split into two Shockley partials, producing stacking faults. Shockley partials nucleate and migrate more easily at interfaces, near the free surface, and in stress-concentration zones, often signaling stacking-fault-mediated slip and even twin initiation. When two symmetric Shockley partials meet on a cross-slip plane, stair-rod dislocations can form. These dislocations are essentially immobile, acting as locks that pin the network and further strengthen hardening. Hirth dislocations arise mainly from dislocation interactions, move poorly, hinder glide, and add to hardening. Frank dislocations, linked to stacking-fault loops, are also immobile. They reshape the local stress field and obstruct passing dislocations. In our study. Their length is generally low, indicating that slip dominates the compression process. The other category (strongly curved segments, short-lived reaction segments, or mixed-character lines) tracks the rapid rearrangement of the network. The increase in this class usually accompanies surface disordering, frequent slips, and local damage.

In this study, all three modes except LRUVC are driven by strong shear strain, which activates many dislocations from the free surface and the Al-Si phase boundary and sends them along the {111} slip-plane family to the next interface. In the FCC system, Shockley partials carry most of the slip. As Shockley lines meet and react during propagation, they generate additional stair-rod dislocations, producing marked local interlocking. The overall fraction of perfect dislocations is low. It is consistent with aluminum’s stacking-fault behavior. Most perfect segments rapidly decompose into Shockley partials, leaving only short remnants at the heads and tails of slip bands. Frank and Hirth dislocations are rare, indicating that climb and stick-slip are largely suppressed under compression. Note that the other class (e.g., entries like in Figure 11(a2) 1/6 [1-14]) mainly reflects high-strain-induced crystal rotation and lattice distortion, which prevent DXA in OVITO from fully resolving the standard cubic frame. When the Burgers vector is rotated back to the cubic basis, many of these segments reassign to stair-rod or Shockley types.

In CC, counts for nearly all dislocation types are highest. The rapid proliferation of dislocations builds a dense network, raising local stress via work hardening. Once interlocking and pinning are overcome, the lengths of different types surge together with strong fluctuations, making this mode the least stable. In UVC, early dislocation nucleation increases. But cyclic loading promotes cross-slip, recovery, and annihilation, which reduce the net build-up of perfect and Shockley segments and shorten the lifetime of stair-rod locks. Consequently, Shockley and stair-rod lengths show a first-increase-then-decrease trend from the mid stage onward. In LRC, stress redistribution by the remelted layer lowers the migration barrier, delaying interlocking in stages. As compression deepens and the remelted layer weakens, its redistribution ability declines, and the accumulation pattern drifts toward CC. A noticeable early population of stair-rod segments in LRC and LRUVC suggests that thermal stress from remelting in the unmelted zone can induce corresponding reaction dislocations.

In LRUVC, nucleation is suppressed at the source, intersections are reduced, and annihilation is accelerated by the synergy between stress redistribution (Generated by the remelted layer) and stress concentration (Generated by ultrasonic vibration). The remelted layer reduces the local effective SFE and the migration barrier, homogenizes stress, and extends the average free path of dislocations. The high-frequency alternating shear of ultrasonics promotes alternating slip and recovery, continuously removing short-range Shockley segments. Remelting-induced stair-rod locks do not readily re-accumulate or persist. With a more uniform force field, local stress peaks and GND density are suppressed, the extension of stacking-fault/twin bands is limited, and the material maintains very low dislocation density, small fluctuations, and a highly stable plastic response throughout loading. Overall, LRUVC avoids the disordered proliferation and network hardening seen in CC. It more effectively suppresses re-accumulation and interlocking than UVC, and achieves stable, process-wide control.

## 4. Discussion

Results of CT experiments showed that there were significant particle debonding, fragmentation, continuous ploughing, and tearing marks on the surface. There are obvious particle displacements, accumulation, and crack propagation on the subsurface. The damage layer is deeper. The average surface roughness of CT reached 0.5336 μm, confirming that extensive particle debonding and interfacial cracking resulted in pronounced surface waviness and deep subsurface damage. Simulations reveal the cause. Hydrostatic stress continues to propagate through the thickness and concentrates at particle-particle contact zones. Both von Mises stress and equivalent plastic strain increase monotonically during loading and form high-strain bands along interface slip systems, which promote interface cracking and particle slip. At the structural and dislocation levels, FCC content drops rapidly at early stages. And defects accumulate. Numerous dislocations are activated, which builds a dense interlocking network and produces marked work hardening through their interactions. These mechanisms show that edge-arc extrusion in CT drives load transfer and work hardening, which in turn causes deep damage and poor surface and subsurface quality. Quantitatively, the CC mode exhibits the highest yield peak stress of 1.84 GPa and maintains a high level with irregular fluctuations, confirming continuous bearing and severe work hardening.

In UVT, the surface shows periodic vibration marks and shallow particle breakage. Ploughing marks are shorter. And the damage layer is markedly shallower. Correspondingly, the surface roughness decreased to 0.2304 μm—about 56.8% lower than CT—indicating that ultrasonic vibration effectively localized deformation to the surface layer and weakened deep interfacial damage, consistent with the simulated attenuation of stress and dislocation density. Simulations indicate that the instantaneous hydrostatic-stress peak at the top is the highest among the modes and launches a downward stress wave. But rapid attenuation leaves the deeper region less affected. Von Mises stress rises quickly in the early stage and then remains at a high level. The equivalent plastic strain exhibits strong strain concentration at the surface and free boundary. Defect statistics show the growth of amorphous regions in high-strain zones and the corresponding drop in FCC content. And ISF/TB counts decline after peaking, reflecting the transformation of stacking faults/twins into disordered phases. Dislocation density surges early. But net accumulation is limited by cross-slip, recovery, and annihilation under cyclic loading. So, the network density is lower than in CT. As a result, UVT confines plastic activity near the surface and disrupts interlocking through periodic loading. However, excess local peaks in the mid-cycle can trigger surface particle fracture and spalling, causing instability in practical machining. Numerically, the UVC peak stress (1.15 GPa) is 37.5% lower than that of CC, and the average rheological stress is reduced by 56.8%, which quantitatively supports the ultrasonic softening effect and the reduction in continuous load bearing.

In LRT, the machined surface is smoother, ploughing marks are thinner, and severe subsurface defects are greatly reduced. Cracks remain visible. But the depth and extent are controlled. Experimentally, the average surface roughness was reduced to 0.0927 μm, an 82.6% reduction compared with CT, indicating that laser remelting effectively suppressed surface waviness and subsurface cracking, which aligns with the simulated dispersion of stress and delayed dislocation accumulation. Simulations indicate that laser remelting converts the near-surface lattice into a disordered solid solution, which disperses stress and strain within the layer, suppresses early defect formation, and limits slip-system activation. As compression reaches the unremelted second layer, the response gradually shifts toward CT, showing that the buffering effect of the remelted layer depends on its thickness. The growth of structural defects is lagging and small. Dislocations are dominated by mild accumulation. Stair-rod/Hirth is finitely generated. Overall, LRT reduces stress concentration and spreads distortion energy, thereby lowering surface and subsurface damage. However, when the indentation depth exceeds the remelted thickness, protection weakens. So, the turning depth must be matched to the heat-affected-layer thickness in practice. This trend is quantitatively reflected by the LRC mode, whose peak stress (0.64 GPa) is 65.2% lower than CC, and average rheological stress decreases by 82.6%. The slow decay and reduced plateau level indicate mild work hardening and stable deformation within the remelted zone.

In LRUVT, experiments achieve the best surface roughness and minimal subsurface damage. Vibration marks become shallow, tearing and particle debonding nearly disappear, the subsurface remains continuous and intact, and cracks are few. The measured roughness reached the lowest value of 0.0642 μm, 72.1% and 30.7% lower than that of UVT and LRT, respectively. This demonstrates that coupling laser remelting and ultrasonic vibration minimizes particle fracture and interfacial defects, producing the smoothest surface and shallowest damage layer, in full agreement with the lowest stress and dislocation levels predicted by simulation. Simulations show that this benefit arises from the coupling of the two energy fields. The initial stress field resembles that of LRT. The remelted layer markedly weakens the ultrasonic stress wave and its peak stress. As compression proceeds, the response smoothly transitions and approaches the stable stage of UVT at larger depth. Throughout loading, the equivalent plastic strain maintains a low peak and a controlled distribution. High-strain localization confines to a minimum region. Structural statistics indicate the lowest levels of stacking faults, the lowest total dislocation content, with only slight fluctuations. And there is almost no large interlocking network, implying that nucleation and interlocking are suppressed at the source. Quantitatively, LRUVC has the lowest initial peak stress (0.60 GPa), 67.4% lower than CC, and the lowest sustained average stress—about 88.0% lower—demonstrating that the combined energy fields achieve the most effective reduction of both peak and steady-state stress. In summary, LRUVT optimizes peak reduction, depth control, and defect suppression through the dispersion buffer of the remelted layer combined with the shallow, rapid plasticization from the ultrasonic, thereby minimizing surface and subsurface damage while preserving removal efficiency. These results confirm the advantage of the composite energy field in both damage control and surface quality. The nearly 70% reduction in peak stress and 90% reduction in average stress quantitatively confirm the advantage of the composite energy field in both damage control and surface quality.

## 5. Conclusions

This paper systematically investigates the damage mechanism of high-silicon aluminum alloy during micro-machining through experiments and molecular dynamics simulations, comparing four processes: conventional turning, ultrasonic vibration turning, laser remelting turning, and laser remelting ultrasonic vibration turning. The research provides a theoretical basis and parameter guidance for the high-quality processing and engineering application of this material. The main conclusions are as follows:

(1) Process comparison shows that conventional turning is prone to cause particle de-adhesion and deep cracks; ultrasonic vibration turning can reduce the damage layer, but there is a risk of surface particle fracture; laser remelting turning can reduce the density of furrows and cracks; while laser remelting ultrasonic vibration turning can effectively suppress tearing, de-adhesion and surface pits, demonstrating the best overall effect.
(2) Molecular dynamics simulations reveal that in conventional turning, continuous loading leads to deep stress transmission, causing significant particle debonding, crack propagation, and dislocation locking, resulting in significant work hardening and severe surface damage. In ultrasonic vibration turning, plastic deformation is confined to the shallow layer, but the transient stress peaks may still cause particle fracture and amorphization. Laser remelting turning homogenizes the stress through the remelting layer, inhibiting the formation of early defects and dislocation networks, resulting in a smoother surface and fewer cracks; however, if the cutting depth exceeds the thickness of the remelting layer, the protective effect weakens.
(3) Laser remelting ultrasonic vibration turning combines the synergistic advantages of remelting layer homogenization and ultrasonic shallow layer plasticity, making the stress distribution more uniform and the peak value lower, minimizing the defect and dislocation connectivity, achieving the best surface and internal integrity, and the highest overall processing quality.

## Figures and Tables

**Table 1:** Experiment parameter.

Processing Mode	Constant (m/min)	Feed Rate (mm/rev)	Turning Depth (mm)	Laser Power (W)	Frequency (kHz)	Pulse Width (ns)
LR	10	0.03	—	15	200	50
CT	10	0.03	0.004	—	—	—
LRT	10	0.03	0.004	15	200	50
UVT	10	0.03	0.004	—	—	—
LRUVT	10	0.03	0.004	15	200	50

**Table 2:** Simulation parameter.

Item	Value/Form	Unit
Box size	140 × 240 × 140	Å
Si diameter/spacing	50/20	Å
Interatomic potential	ADP	—
Equilibration ensemble	NPT	—
Equilibration temp	300	K
Heat source radius	25	Å
Heat source energy	943.5	eV
Heat-up time	80	ps
Cooling time	480	ps
Compression ensemble	NVE	—

## Data Availability

The original contributions presented in the study are included in the article, further inquiries can be directed to the corresponding author.

## Abbreviations

The following abbreviations are used in this manuscript:

PRMMCs	Particle-reinforced metal matrix composites
LR	Laser Remelting
CT	Conventional Turning
UVT	Ultrasonic Vibration Turning
LRT	Laser Remelting Turning
LRUVT	Laser-Remelting Ultrasonic-Vibration Turning
CC	Conventional Compression
UVC	Ultrasonic Vibration Compression
LRC	Laser-Remelting Compression
LRUVC	Laser-Remelting Ultrasonic-Vibration Compression
MD	Molecular Dynamics
LAMMPS	Large-scale Atomic/Molecular Massively Parallel Simulator
OVITO	Open Visualization Tool
ADP	Angular-Dependent Potential
EAM	Embedded-Atom Method
CAT	crystal analysis tool
DXA	Dislocation Extraction Algorithm
FCC	Face-Centered Cubic
ISF	Intrinsic stacking faults
TB	Twin boundary
